# Epidemiology of Suicide/Suicide Attempt and Its Association with Individual, Family, and Social Factors in Eastern Part of Iran: A Historical Cohort Study

**Published:** 2019-08

**Authors:** Ali ALAMI, Mahbobeh NEJATIAN, Elaheh LAEL-MONFARED, Alireza JAFARI

**Affiliations:** 1. Department of Public Health, School of Public Health, Gonabad University of Medical Sciences, Gonabad, Iran; 2. Social Determinants of Health Research Center, Gonabad University of Medical Sciences, Gonabad, Iran; 3. Student Research Committee, School of Public Health and Safety, Shahid Beheshti University of Medical Sciences, Tehran, Iran

**Keywords:** Completed suicide, Risk factor, Cohort, Iran

## Abstract

**Background:**

Suicide/suicide attempt, as a major public health problem, has been included among anti-social behaviors. We aimed to investigate the epidemiology of suicide and some associated individual, family, and social factors.

**Methods:**

A historical cohort study was conducted among all cases (748 persons) reports related to suicide/suicide attempt that register in hospital reporting system and health care center in Gonabad and Bajestan, Iran, from 2009 to 2014. The required data were extracted through a checklist. Descriptive (mean and standard deviation) and analytic statistics (*t*-test, Chi-square, and logistic regression) applied to analyzed data. All data were analyzed using SPSS software.

**Results:**

Of the 748 reported suicide attempters, 17 cases (2.3%) had completed suicide. The annual average incidence rate of suicide was 110.03 per 100,000 populations. The mean age of suicide attempt had significantly decreased during the time (*P*=0.007). Furthermore, a significant association was observed between outcome of suicide and place of residence (*P*=0.019), history of physical illnesses (*P*=0.002), and method of suicide (*P*=0.001).

**Conclusion:**

Due to trend of age among suicide attempters, considering intervention programs of suicide prevention for school pupils and university students especially individuals residing in rural areas, as well as individuals suffering from physical illness would be effective to reduce the rate of suicide.

## Introduction

Suicide is recognized as a deliberate act of self-harm leading to death (1). Suicide/suicide attempt, as anti-social behavior, also is a major public health problem as well as one of the most important indicators of public mental health (2). Suicide rate in Iran has been also lower than that in most countries especially in Western societies, but such a value has been higher than that reported among nations located in the Middle East (3). Suicide is the third leading cause of mortality among individuals aged 21 to 30 yr old in some countries (3). Besides, research studies conducted in different continents have shown that suicide attempts are on a rise and the statistics associated with successful suicide attempts in recent years have been so worrying. Suicide is even counted as social harm in a way that suicide attempt is oriented towards harming oneself consciously especially among more anxious, aggressive, or disabled self-oriented people failed in establishing social relationships (3).

Most individuals committing suicide have had suicide attempts (3) so that each suicide attempt can increase the rate of successful suicide risk by 32% (4). In fact, suicide attempts are more common among young women and more serious in older men (5). Therefore, comparing suicide rate among men and women has suggested that women are at the risk of suicide more than men. According to the statistics released in this respect, the rate of suicide attempts in women is 3 to 4 times more than men and such figures have been significantly higher in Latin America and Northern Europe. The number of suicide attempts are 8 to 10 times higher than successful cases of suicide (6).

Researchers have similarly cited several factors associated with suicide attempts in a way that investigations have revealed that people with suicide attempts are inflicted with diagnosable mental problems. Moreover, 60% of those committing suicide have had a history of mental illnesses in the past and the probability of suicide attempts in such patients has been 3 to 12 times more than that in ordinary individuals. Likewise, 40% of people suffering from depression have had a history of suicide attempts (7). Psychiatric disorders particularly depression and substance abuse have been also among the most important underlying causes of suicide attempts (7).

Moreover; in terms of the concurrency of psychiatric disorders and suicide attempts, the maximum amounts have been reported for depression as well as antisocial and mood disorders (8). Suicide attempters have five major characteristics including serious problems with their marital partner, presence of a new person in life (second spouse), presence of a disease in a family member, individual’s serious physical illness, and emotional pain (9). Moreover, people who commit suicide have a history of chronic problems associated with marriage, children, job, financial issues, health, and addiction (9). Stressful life events could be seen more frequently in suicide attempts (10). Heknin et al., for instance, examining life events before suicide attempts identified separation, divorce, serious family disputes, financial difficulties, work-related problems, and unemployment in younger age group and also physical illnesses and retirement in the elderly as life events (10). Meanwhile, there were some potential risk factors of suicide attempts in Iran including young age, femininity, history of psychiatric illnesses, alcohol use and smoking, and unemployment (10).

Recognizing potential factors concerning suicide especially in counties with young population such as Iran would be very important. Besides, despite an extensive search, we could not find studies on suicide have been in the East of Iran. Therefore, we aimed to investigate the epidemiology of suicide and some associated individual, family, and social factors.

## Methods

A historical cohort study was conducted among individuals who live in Gonabad and Bajestan, Eastern Iran with roughly 120,000 populations from 2009 to 2014. We used all cases (748 persons) reports related to suicide/suicide attempt that register in hospital reporting system and health care center in Gonabad and Bajestan, Iran. The required data were extracted through a checklist. The inclusion criteria included participants who were willingness to participate in this study and lived there. The participants were excluded if their clinical and demographic information were incomplete and they were unwillingness to participate.

We gathered independent variables including age, gender, marital status, residency location, occupation, level of education, suicide method, history of physical and mental illness, as well as history of previous suicide. In this study, outcome of suicide (complete/incomplete) was considered as dependent variable and univariate and multiple logistic regression models were conducted to examine the relationship between suicide statuses with individual characteristics, family and social parameters. In terms of the regression analysis, the variables were initially entered into univariate regression and then those with a significant level lower than 0.2 were fed into multiple regression. All data were analyzed by the SPSS software (ver. 18, Chicago, IL, USA) and Significance level was set at 0.05 in all the tests.

## Ethical approval

This study was approved by Gonabad University of Medical Sciences Ethics Committee (IR.GMU.REC.1394.37). All participations were assured that all the information would be kept confidential and would not be revealed unless for research purposes and in an anonymous form. Participants were allowed to decline participation at any stage of research.

## Results

**Fig. 1:**
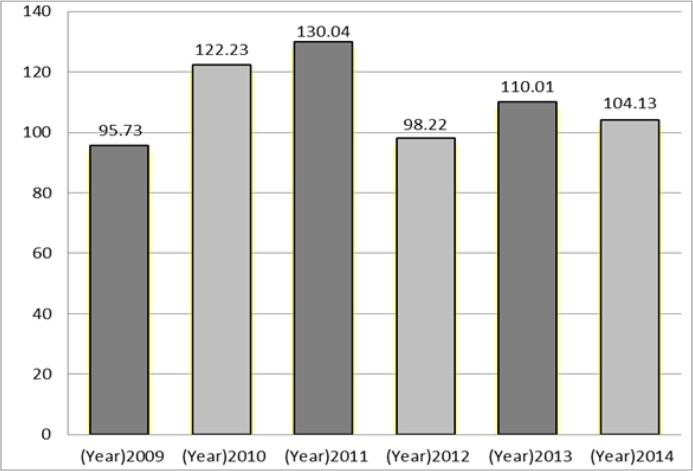
Incidence rate of suicide in different years

There were 748 suicide attempt cases [women= 457(61.2%), men = 290(38.8%)]. The number (percent) of completed suicides was 17 cases (2.3%) in which 71% were male. The mean age (SD) of the suicide attempters was 26.37 (10.82) with the age range between 13 and 84 yr. The incidence rate of suicide/suicide attempt per 100,000 populations presents in Fig. 1.

Demographic characteristics of suicide attempters and their methods of suicide are shown in Table 1. Association between characteristics of the participants and the outcomes of suicide are presented in Table 2. As seen in Table 3, the mean age of individuals committing suicide had significantly reduced between 2009 and 2014 (*P*=0.007). Poisoning was the most common method of suicide, which was significantly higher in women (n=436, %=64.98) population in comparison with men (n=235, %=35.02) (*P*<0.001). Likewise, our results showed suicide outcomes were significantly associated with living place (*P*=0.019), history of physical illnesses (*P*=0.002), and suicide method (*P*=0.001) (Table 4).

**Table 1:** Demographic characteristics of suicide attempters and methods of suicide in terms of years (2009–14)

***Variables***	***Description***		***Year***					
			***2009***	***2010***	***2011***	***2012***	***2013***	***2014***
Sex	n (%)	Male	38(31.7)	34(27.2)	32(28.8)	69(46.9)	71(51.8)	50(46.3)
		Female	82(68.3)	91(72.8)	79(71.2)	78(53.1)	66(48.2)	58(53.7)
Marital status	n (%)	Married	80(66.7)	83(66.4)	62(55.9)	94(63.9)	78(56.9)	65(60.2)
		Single	40(33.3)	42(33.6)	49(44.1)	53(36.1)	59(43.1)	43(39.8)
Residency location	n (%)	Urban	91(75.8)	88(70.4)	71 (64)	103(70.1)	105(76.6)	69(63.9)
		Rural	29(24.2)	37(29.6)	40(36)	44(29.9)	32(23.4)	39(36.1)
Occupation	n (%)	Governmental	23(19.2)	30(24)	21(18.9)	42(28.6)	37(27)	35(32.4)
		Housewife	49(40.8)	56(44.8)	52(46.8)	50(34)	52(38)	48(44.4)
		Student	34(28.3)	30(24)	27(24.3)	29(19.7)	15(10.9)	10(9.3)
		Unemployed	8(6.7)	4(3.2)	9(8.1)	20(13.6)	25(18.2)	14(13)
		Other	6(5)	5(4)	2(1.8)	6(4.1)	8(5.8)	1(0.9)
Level of education	n (%)	Illiterate	4(3.3)	4(3.2)	5(4.5)	8(5.4)	6(4.4)	11(10.2)
		Primary	8(6.7)	20(16)	4(3.6)	18(12.2)	18(13.1)	12(11.1)
		Secondary	33(27.5)	20(16)	25(22.5)	50(34.1)	49(35.8)	49(45.4)
		High school	57(47.5)	62(49.6)	57(51.4)	55(37.4)	56(40.9)	34(31.4)
		Academic	18(15)	19(15.2)	20(18)	16(10.9)	8(5.8)	2(1.9)
Suicide method	n (%)	Poisoning with Drug	99(82.5)	101(80.8)	76(68.5)	99(67.3)	102 (74.5)	74(68.4)
		poisoning with Other substances	4(3.3)	1(0.8)	11(9.9)	28(19)	14(10.2)	19(17.6)
		Eating poison	3(2.5)	12(9.6)	10(9)	2(1.4)	3(2.2)	3(2.8)
		Self-injury	8(6.7)	6(4.8)	11(9.9)	10(6.8)	16(11.7)	6(5.6)
		Self-Burning	2(1.7)	3(2.4)	2(1.8)	5(3.4)	1(0.7)	3(2.8)
		Hanging	4(3.3)	2(1.6)	1(0.9)	3(2)	1(0.7)	3(2.8)
History of physical illness	n (%)	YES	3(2.5)	5(4)	8(7.2)	7(4.8)	1(0.7)	2(1.9)
		NO	117(97.5)	120(96)	103(92.8)	140(95.2)	136(99.3)	106(98.1)
History of mental illness	n (%)	YES	26(21.7)	9(7.2)	8(7.2)	11(7.5)	6(4.4)	9(8.3)
		NO	94(78.3)	116(92.8)	103(92.8)	135(92.5)	131(95.6)	99(91.7)
History of suicide	n (%)	YES	20(16.7)	6(4.8)	9(8.2)	10(6.8)	2(1.2)	17(15.7)
		NO	100(83.3)	119(95.2)	101(91.8)	137(93.2)	135(98.8)	91(84.3)
Outcome of suicide	n (%)	Complete	7(5.8)	1(0.8)	3(2.7)	3(2)	1(0.7)	2(1.9)
		Incomplete	113(94.2)	124(99.2)	108(97.3)	144(98)	136(99.3)	106(98.1)

**Table 2:** Relationship between characteristics of participants and outcome of suicide

***Variables***		***Outcome of suicide***		P-value^*^
		***Complete n (%)***	***Incomplete n (%)***	
Sex	Male	12 (4.1)	278 (95.9)	0.008
	Female	5 (1.1)	453 (98.9)	
Marital status	Married	13 (2.8)	449 (97.2)	0.207
	Single	4 (1.4)	282 (98.6)	
Residency location	Urban	7 (1.3)	520 (98.7)	0.007
	Rural	10 (4.5)	211 (95.5)	
Occupation	Governmental	8 (4.3)	180 (95.7)	0.108
	Housewife	5 (1.6)	302 (98.4)	
	Other	4 (1.6)	249 (98.4)	
Education	Non-academic	16 (2.4)	649 (97.6)	0.710
	Academic	1 (1.2)	82 (98.8)	
History of mental illness	Yes	6 (8.7)	63 (91.3)	0.003
	No	11 (1.6)	667 (98.4)	
History of physical illness	Yes	4 (15.4)	22 (84.6)	0.002
	No	13 (1.8)	709 (98.2)	
History of suicide	Yes	2 (3.1)	62 (96.9)	0.650
	No	15 (2.2)	668 (97.8)	
Suicide method	Non-physical	7 (1.1)	654 (98.9)	0.001
	Physical	10 (11.5)	77 (88.5)	

2* χ

**Table 3:** Mean of the results for the analysis of variance (ANOVA) associated with suicide attempters in general and in terms of gender

***Years***	***Age***		***All***
	***Male***	***Female***	
2009	30.88 ±13.67	27.48 ±12.60	29.06 ±13.15
2010	30.85 ±11.92	23.38 ±7.34	27.25 ±10.63
2011	30.25 ±11.89	24.73 ±10.45	27.29 ±11.44
2012	29.03 ±15.41	22.98 ±7.49	24.67 ±10.61
2013	28.64 ±11.67	23.97 ±8.47	25.20 ±9.59
2014	24.24 ±7.80	24.77 ±8.99	24.61 ±8.61
*P*-value	0.120	0.091	0.007

**Table 4:** Results of univariate and multiple regression analysis in terms of suicide and associated individual, familial and social factors among participants

***Variables***		***Results of univariate regression***			***Results of multiple regression***		
		***OR***	***95 % C.I***	***P-value***	***OR***	***95 % C.I***	***P-value***
Age		1.031	0.99–1.06	0.075	1.010	0.969–1.053	0.639
Gender		3.911	1.36–11.21	0.011	2.023	0.582–7.35	0.268
Marital status		0.490	0.15–1.51	0.216	-	-	-
Residency location		3.521	1.32–9.37	0.001	3.883	1.255–12.014	0.019
Level of Education	Illiterate	2.212	0.135–36.403	0.577	-	-	-
	Primary	3.195	0.325–31.374	0.319	-	-	-
	Secondary	1.855	0.214–16.118	0.575	-	-	-
	High school	1.828	0.222–15.068	0.575	-	-	-
History of mental illness		5.775	2.06–16.13	0.001	2.501	0.740–8.449	0.140
History of physical illness		9.916	2.99–32.86	0.001	10.307	2.386–44.516	0.002
History of suicide		1.437	0.32–6.42	0.636	-	-	-
Method of suicide		12.134	4.48–32.79	0.001	7.324	2.307–23.254	0.001

## Discussion

This study conducted to recognize potential risk factors of suicide/suicide attempts and their association with outcome of suicide. We found that mean age of the suicide attempters significantly decreased between 2009 and 2014. The results of multiple regression analysis also showed a significant relationship between place of residence, history of physical illnesses, as well as method of suicide and outcome of suicide. Besides, we did not find significant association between outcome of the participants’ suicide and their age, sex, and history of mental illnesses.

In the present study, the incidence rate of completed suicide was 2.3%. There were different rates in various studies from 3.2% to 13.1% (11–17). The annual suicide rate was also equal to 110.03 (per 100,000 population). Incidence rate of suicide attempt was reported to be 92.5%, 90%, and 60.2% in the research studies (17–19), respectively. According to the comparison, although there was a higher rate of suicide attempts in Gonabad and Bajestan than in other regions, completed suicide is lower in these districts. Our finding showed that 8.5% of the participants had a history of prior suicide. This value also varied from 2.5% to 34% in different investigations (8, 10, 15, 18, 19).

Furthermore, the most commonly used method of suicide was medication poisoning that was consistent with the findings of several studies (2, 3, 8, 13, 17, 18, 20).

A number of other investigations also reported some other common methods (14, 21, 22), at the reason for the prevalence of committing suicide via medications could be due to its availability and awareness of its safer consequences compared to more invasive methods.

According to our results, the mean age of suicide attempters had significantly decreased which was similar to the findings of other studies in this respect (13, 20, 23, 24). The high incidence rate of suicide among the youth is also considered as a dilemma originating from social, cultural, familial, and economic abnormalities. Moreover, presence of probable factors such as despair, self-esteem disorders, frustration, lack of understanding, inappropriate behaviors from parents and others, and unemployment all can be taken into account as the sources of mental and psychological tension among the youth which finally lead to consequences such as suicide (13).

Result of the present study also showed a significant association between outcome of suicide and the attempters’ place of residence. Completed suicide had occurred at higher rates in rural than in urban areas. This result was similar to the findings of several studies (12, 25, 26), however, they contradicted the findings obtained in some related investigations (11, 25–29). This inconsistency could be due to differences in study population and increased migration from rural to urban areas in the contexts examined. Likewise, the higher rate of suicides in rural areas could be justified through some socio-economic and cultural factors such as unemployment, poor economic status, and low education level of individuals.

In this study, a significant association was also observed between history of physical illness and outcome of suicide which was comparable with study results (30), but in conflict with those reported in another investigation (31). The major factors affecting suicide were diseases such as epilepsy, diabetes, AIDS, cancer, high blood pressure, lung diseases, spinal cord injuries, premenstrual syndrome, and rheumatoid arthritis (17). The reason for the given differences in the results of studies in the related literature would be due to prevailing culture in the research environments, endemic diseases specific to each region, and various types of statistical methods used in different studies compared with those employed in the present study. More investigations to clarify this ambiguity is recommended. Our finding revealed a significant relation between outcome of suicide and method of suicide that is completed suicide had happened among individuals using violent physical methods. This was in line with many study results (23, 28, 31). Naturally, using more violent methods such as hanging, self-immolation, or use of firearms and cold ones are more likely to be fatal; so, there is a higher rate of completed suicide in these individuals.

Based on the results of this study, there was no statistically significant association between age and outcome of suicide which were consistent with other findings (25) and in conflict with the results of studies (12). One of the probable cause for this difference would be due to statistical method used in the present study, which controls the potential confounders.

According to our results, there was no significant association between sex and outcome of suicide which was in line with several studies (3, 25), and in contrast with the findings of a number of other studies (11, 13, 23, 24, 30). The differences in this respect could be due to gender composition and different cultural issues prevailing among the study populations. There could also be a statistical reason to interpret this difference. Some researchers compare outcome of study between male and female without control possible confounding factors (11, 23, 30). While, in our study, we applied multiple regression to control confounding effects. Therefore, to determine real association between suicide and sex, use of statistical modeling is recommended.

The results obtained in the present study also showed no significant relationship between history of mental illnesses and outcome of suicide which was in agreement with the findings of some studies (23, 31); but in conflict with the findings of investigations conducted by other studies (3, 7, 11, 30). One potential reason for the difference could be that we used suicide-reporting data. Due to the nature of mental illness and difficulty in properly diagnosing them, the data may not be recorded with high precision. Besides, such an inconsistency could be due to different attitudes towards individuals suffering from mental illnesses within societies by which such people could be treated like other patients or excluded from society and finally attempt suicide due to loneliness, isolation from society, and severe depression. Furthermore, people with mental disorders have lower levels of life expectancy and greater aggressiveness compared to other groups. Sometimes this aggression targets others and sometimes oneself. Such individuals also have low levels of self-esteem and poor problem-solving skills; thus, they have lower threshold for tolerance and act impulsively.

Like any other investigations, the present study had its own strengths and limitations. Paying attention to use the data of suicide reporting system, checking all recorded and reported cases of suicide/suicide attempts, the research methodology adopted, and use of statistical modeling would be some strengths of this study. On the other hand, investigating data in reporting systems has its own limitations. The available data, for instance, would not represent all suicide cases. There was also the possibility not to consider some important and effective variables in the given recording systems. Therefore, it was recommended to do concurrent cohort studies in order to overcome these disadvantages.

The results of this study can be used by policy makers, health service delivery systems, counseling centers, and social emergency centers to plan and implement preventive interventions. According to the results of the multiple analysis based on marital status, it was suggested to take into account the potential confounding variables in the analysis of suicide.

## Conclusion

There is a significant association between outcome of suicide and place of residence, history of physical illnesses, and method of suicide. Meanwhile, due to the reduced mean age of suicide attempters, paying more attention to young age would be effective to reduce incidence rate among community.

## Ethical considerations

Ethical issues (Including plagiarism, informed consent, misconduct, data fabrication and/or falsification, double publication and/or submission, redundancy, etc.) have been completely observed by the authors.
